# Wearable health monitoring: wave of the future or waste of time?

**DOI:** 10.21542/gcsp.2024.21

**Published:** 2024-04-20

**Authors:** Nicholas L. Hudock, Hunter Hughes, Nour Shaheen, Abdelraouf Ramadan, Kinna Parikh, FNU Anamika, Rohit Jain

**Affiliations:** 1 Penn State College of Medicine, Hershey, PA, USA; 2 Faculty of Medicine Alexandria University, Al Attarin, Alexandria, Egypt; 3 Faculty of Medicine, Helwan University, Helwan, Egypt; 4 Western Reserve Health Education, Ohio, USA; 5 University College of Medical Sciences, New Delhi, India

## Abstract

Background: Atrial fibrillation is responsible for over 400,000 hospitalizations in the United States (US) each year. This costs the US health system over 4 billion each year. New smartwatches can constantly monitor pulse, oxygen saturation, and even heart rhythm. The FDA has provided clearance for select smartwatches to detect arrhythmias, including atrial fibrillation.

Findings: These devices are not currently widely implemented as diagnostic tools. In this review, we delve into the mechanism of how smartwatches work as healthcare tools and how they capture health data. Additionally, we analyze the reliability of the data collected by smartwatches and the accuracy of their sensors in monitoring health parameters. Moreover, we explore the accessibility of smartwatches as healthcare tools and their potential to promote self-care among individuals. Finally, we assess the outcomes of using smartwatches in healthcare, including the limited studies on the clinical effects and barriers to uptake by the community.

Conclusion: Although smartwatches are accurate for the detection of atrial fibrillation, they still face many hurdles, including access to aging populations and trust in the medical community.

## Introduction

Cardiac arrhythmia is a variation of the normal heart rate and/or rhythm that is not physiologically justified^1^. The mechanisms responsible for cardiac arrhythmias are generally divided into two major categories: abnormal impulse formation and conduction disturbances^1^. Atrial fibrillation (AF) is the most common arrhythmia. The worldwide prevalence of AF is 37.6 million cases, approximately 0.5% of the global population, and future projections suggest that the absolute atrial fibrillation burden may increase by over 60% by 2050^2^. A new or suspected diagnosis of AF or other cardiac arrhythmia in an otherwise healthy patient does not come without cost, whether from outpatient physician visits or management with rate control, rhythm control, or anticoagulation drugs. However, failure to detect and diagnose AF or other arrhythmias can lead to far more dire and expensive complications such as cerebrovascular accidents (CVA). In a single year in the US, AF was estimated to be responsible for 599,790 emergency department visits, 453,060 hospitalizations, and 21,712 deaths. Hospitalizations alone are estimated to cost approximately 4 billion US dollars^3^.

Smartwatches have become a commonly-owned item among American consumers and have gained popularity abroad. The number of smartwatch and fitness-tracker shipments is expected to increase by 11% between 2021 and 2024. It is expected that 280 million units will be shipped worldwide by 2024^4^. While a smartwatch is really any mobile wrist device with a screen to use instead of a phone, modern smartwatches go far beyond simply calling and texting. They now incorporate the features of a phone, personal assistant (Siri, etc.), and even fitness and health tracking. Common smartwatch brands in the US include Apple, Android (Samsung), Fitbit, and Garmin. Collectively, Apple and Android dominate, accounting for approximately 98% of the market^4^. In fact, Apple shipped more watches in the first quarter of 2016 than the entire Swiss watch industry, including manufacturers such as Rolex and Swatch^4^. The increased prevalence of smartwatches in America has led to a plethora of newly available biometric data for healthcare professionals in the management of patients. With just a smartphone and a few basic apps, patients have been able to track information such as steps, activity, and blood pressure, or even provide a basic medical ID to physicians in case of an emergency^4^. Smartwatches are able to connect to this phone data, and with additional sensors, they have the ability to track important health features such as activity intensity or specific exercises by monitoring vitals such as heart rate, respiratory rate, heart rhythm, oxygen saturation, and even blood pressure. More information regarding FDA-approved smartwatches for health monitoring is available in Table 1.

**Table 1:** Comparison of available FDA cleared health monitoring watches. Watch stats shown are common marketing stats for functionality and useability beyond health monitoring functions.

	Fitbit Sense	Apple Watch Series 7	Samsung Galaxy Watch 4	Withings Scanwatch
Cost	$249.95	$399	$199	$279
Charge	6 days	18 h	40 h	30 days
Water proof	Yes	Yes	Yes	Yes
Pulse monitor	Yes	Yes	Yes	Yes
O2 saturation	Yes	Yes	Yes	Yes
ECG capabilities	Yes	Yes	Yes	Yes
Voice assistant	Google/Alexa	Siri	Bixby	No
Workout tracking	Yes	Yes	Yes	Yes

At the intersection of health care and technology, we have the opportunity to advance the standards of practice to incorporate the health data available through consumer-owned technology. If personal devices were successfully integrated into healthcare, patients would gain an accessible, noninvasive alternative to traditional monitors and have peace of mind knowing that their health is continuously monitored. The level of integration and limits of current technology may be the only barrier to this bright future. In this review, we summarize the mechanism and current state of accessibility, usability, and patient outcomes of smartwatch health monitoring.

## Mechanism

In the inpatient setting, vital signs and cardiac function were monitored using a multilead ECG, pulse oximetry device, and blood pressure cuff. The mechanism for the compression of data, interpretation, and the visual generator that creates the ECG with which we are familiar has been described^5^. The basic concept of ECG monitoring is the attachment of electrodes to the body and visualization of electrical signals produced by the heart^6^. As the heart depolarizes to contract, a wave of electrical signals is conducted, as it repolarizes the electrical signal moving in the opposite direction. Modern blood pressure cuffs utilize a vibration sensor in a pneumatic cuff to occlude an artery before detecting systolic and diastolic blood pressures upon the return and cessation of blood vibrations through the vessel^7^. The pulse oximetry device measures oxygen saturation by illuminating the skin and measuring changes in the light absorption of oxygenated and deoxygenated blood at two light wavelengths: 660 nm and 940 nm^8^. The ratio of absorbance at these wavelengths is calculated and calibrated against standardized measurements of arterial oxygen saturation to establish the pulse oximeter’s measure of arterial saturation^8^.

Current smartwatches obtain pulse and pulse oximetry monitoring data through photoplethysmography (PPG). PPG utilizes a light source and photodetector to determine changes in light intensity on the surface of the skin, correlating to changes in blood tissue volume during different phases of the cardiac cycle^9^. In practice, PPG can be split into two data sources: a quasi-static direct current (DC) component, which represents light reflected/transmitted from static arterial blood, venous blood, skin, and tissues; and a pulsatile alternating current (AC) component, which arises from the modulation of light absorption due to changes in arterial blood volume^10^. By taking thousands of measurements per second, the watch can detect these changes and calculate the pulse rate, blood flow, and oxygenation.

Technology companies have attempted to expand the current flow monitoring system to incorporate blood pressure measurements. In these cuffless blood pressure monitoring systems, the user must first calibrate the PPG system piece (watch or phone) by using a blood pressure cuff linked to the device. Serial blood pressures are measured and analyzed against the PPG-calculated blood flow and this data can then be used to extrapolate blood pressure at a later time, based only on PPG^11^. Thus, once a patient has a baseline pressure calibrated with the watch, they are able to instantly estimate blood pressure using heartbeat and photoplethysmography wave analysis.

**Figure 1.**
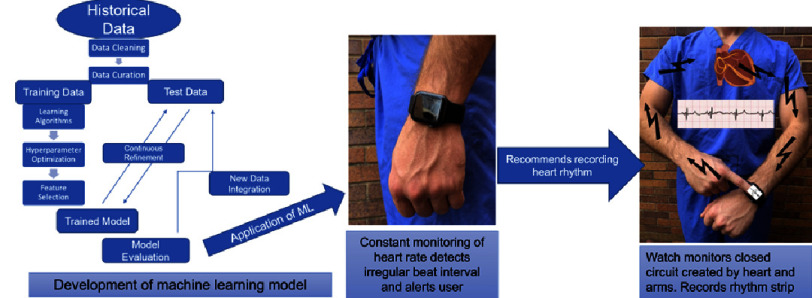
Deriving the single lead ECG for Apple Watch. Illustration of Apple Watch heart rate and rhythm monitoring. Developers trained machine learning models to passively monitor heart rate for irregularities, and alert users. When alerted recordings of heart rhythm can be taken by creating a looping circuit through the watch.

Smartwatches employ an adaptation on the single lead ecg using electrical monitors and PPG data to monitor the hearts rhythm. During regular wear, the electrode and PPG monitor the heart rate and electrical signal, and if the watch suspects that the user may be undergoing an abnormal heart rhythm, it will notify the user and instruct the user to perform ECG tracing. Users wearing a smartwatch are typically instructed to place the pad of the second digit on the opposite hand on the face of the smartwatch. Single ECG monitoring is performed by creating a closed electrical loop created by the watch, arm, chest, arm, watch circuit, and pulse monitoring^12^. This process is illustrated in Figure 1. Algorithms have been created and described to interpret this single-source electrical activity into heart rate and rhythm, and the basis of machine learning model development is summarized in Figure 1^13^.

## Overview of accessibility

Approximately 20 million smartwatches were sold in the US in 2019, meaning nearly 1 in 10 US adults bought a smartwatch in just that year. According to Pew Research Center, 21% of US adults regularly wear a smartwatch or wearable fitness tracker. The data shed interesting light on the socioeconomic distribution of wearables, as 31% of households earning more than $75,000 wear a smartwatch or fitness tracker as compared to 12% of those whose annual household income falls below $30,000^14^. The price of FDA-cleared smartwatches for monitoring health data ranges from approximately $199 for the entry level Samsung, to $400 for the most advanced Apple Watch, with many options in between^15,16^. This is a one-time cost and does not come with any subscription fees; the functions work for the lifetime of the watch. It is worth noting, however, that smartwatches require a phone to record their data, which is not accounted for in the initial costs. Compared with the daily costs of monitoring with a device such as a Holter monitor or implantable monitor, there is an opportunity for significant savings if smartwatches are integrated into the healthcare system. One notable limitation of this comparison is that smartwatches are currently only cleared for detecting AF, whereas Holter’s and implantable monitors may detect other arrhythmias such as ventricular tachycardia or heart blocks.

According to Hillrom billing and coding from 2020, Medicare could be billed $92 for every 48-hour period of continuous monitoring by an external device accompanied by analysis by a physician^17^. In a cost-benefit analysis of single-interval Holter monitoring, Lenane et al. found that only 15% of the subjects monitored attained a diagnosis of AF without any additional medical events or testing^18^. Meanwhile 11% underwent repeated testing despite no clinical events and reached no diagnosis. 13% of patients suffered adverse clinical events despite reaching no diagnosis with the Holter monitor^18^. To achieve continuous monitoring, implantable cardiac monitoring is required. This incurs an estimated cost of approximately $6500 for placement, and average monthly monitoring costing around $58^19^. It was still, on average, $805 cheaper to monitor patients with possible AF in this way as opposed to prophylactically placing all patients on anticoagulation^19^. The need for a diagnostic tool for patients that is not physically burdening, yet able to provide continuous monitoring, has driven the plethora of research in examining the validity of smartwatches as health care tools.

FDA clearance of smartwatches to function as single-lead ECG’s may have led them to starting to function as stand-alone diagnostic tools. One of the most promising trials in terms of clinical accessibility and validity is the REACT-AF trial. This is sponsored by the American Heart Association and is studying whether the Apple Watch may help guide treatment for time-delimited anticoagulation and reduce the incidence of bleeding^20^. However, this FDA clearance is only provided for “wellness” technology, aimed at expediting the application of perceived low-risk technology to medicine and medical research. They remain not a standard of care, but a luxury, and this itself complicates their adoption as a medical device and the future of insurance coverage for patients in need of financial assistance. Despite the lack of insurance coverage for an item such as a smartwatch, current incentives through specific insurance plans allow individuals to acquire a free or reduced-price smartwatch. Specific programs like “Attain” by Aetna, “Motion” by United Health Care, or “Vitality plus” membership with John Hancock allow patients to obtain and work, or rather work-out, off the price of the watch^21–23^. Determination of the medical necessity or expansion of such programs could provide a means to patients with lower socioeconomic or physical functional status to acquire a smartwatch. Data from the implementation of these programs could serve insurance or single payer policy of non-US governments in cost-benefit analysis to provide free or reduced-cost smartwatches to citizens in need.

One concern in the use of wearable technology for monitoring health data is user ability and the adoption of the technology. In a survey of 600 senior citizens who were aware of wearable technology devices, 75% self-reported they had “low experience” using items such as a smartwatch^24^. In a focus group study of 19 senior citizens interested in the features and benefits of a smartwatch, concerns were raised regarding usability, including accessibility (larger icons), notification customization, and intuitive interface design (unambiguous icons and assessment scales)^25^. In a review by Ehrler et al., it was concluded that while smartwatches have the potential to be close assistants to the elderly, this cannot be achieved without dedicating significant effort to designing appropriate user interfaces and dedicated hardware to respond to the constraints associated with potential physical and cognitive impairments^26^. Finally, it cannot be discounted that access to health data has the potential to influence the levels of medical anxiety. A study by Filppaios et al. determined that wearing a smartwatch and receiving health alerts was not significantly associated with changes in self-reported anxiety, but that it did significantly reduce patients perception of physical health status^27^. As more tech savvy Gen X and Millennials age, the accessibility of smartwatches by the elderly may increase, changing the usefulness of these devices without the development of new interfaces.

One major limitation of current smartwatch-monitored biometric data is its availability to physicians. Currently, there are only a small number of programs or apps that interface directly with electronic medical records. Programs such as Microsoft HealthVault are available but require patients to upload their own data, and the development of programs that provide automatic linking of health data to electronic medical records requires a long and expensive process of FDA approval.

## Reliability and outcomes

Perhaps the most important question is “can current market wearable technology compare to traditional medical device monitoring as a diagnostic tool?” In one of the largest population studies, Perez et al. monitored over 400,000 participants who self-reported no previous diagnosis of AF and wore an Apple Watch with ECG capability daily^28^. Of these participants, 2161 received a notification of irregular pulses, 450 returned ECG patches that could be analyzed, and AF was detected in 34% of these patches^28^. Among those who received a notification of an irregular pulse while using the Apple Watch ECG, 84% of irregular pulse notifications were consistent with AF^28^.

A similar large-scale investigation was conducted in China, studying over 187,000 individuals who wore a Huawei Watch GT smartwatch; 424 individuals received notifications of irregular rhythm, 262 followed up with their health provider, and 227 (87%) were diagnosed with AF^29^. While this study utilized a large population, these watches are not common in the US as Huawei telecoms are banned in the US. In a smaller sample independent review comparing the accuracy of Apple Watches ECG to traditional 12 lead ECG monitoring, cardiologists found the Apple Watch to be 96% sensitive and 91% specific in the detection of atrial fibrillation, and 25% sensitive and 99% specific for atrial flutter^30^.

Wasserlauf et al. monitored 24 patients with a history of paroxysmal AF who wore both an Apple Watch with a KardiaBand™ (AliveCor) and who had an internal cardiac monitoring device. They found the Apple Watch to be 97.5% sensitive to episodes of AF, determined by irregular pulse notification, and comparison of recorded ECG with the KardiaBand^31^.

In a study using Samsung smartwatches, 508 inpatients were analyzed using both Samsung smartwatches and traditional ECG. The Samsung watch was found to be 94% sensitive and 98% specific for detecting AF^32^.

Interestingly, an assessment of the accuracy of the Apple Watch and Fitbit against a Holter monitor found that in ambulatory patients, there were significant degrees of underestimation of AF episodes when the heart rate increased^33^. Koshy et al. conducted a similar comparison and found significant heart rate underestimation in episodes of AF, but that the Apple Watch was more accurate than the Fitbit^34^.

Preliminary trials of the Withings Scanwatch have examined its use in detecting elongations in QT interval, rather than detecting AF. The Scanwatch was found to be 91% accurate at detecting a long QT syndrome (>460 ms QT)^35^. Despite an increasing body of literature on the accuracy of smartwatch ECGs, relatively few studies have examined whether smartwatch ECGs improve arrhythmia patient outcomes. These studies are summarized in Table 2.

**Table 2:** Analyses of smartwatch ECG accuracy. Studies had differing primary objectives, and examined different watches all outlined above.

**Source**	**Watch**	**Primary Objective**	**Particpants**	**Outcome**
*Perez et al.*	Apple Watch	Detection of AF after irregular pulse notification	400,000	PPV (0.84) of AF on apple ECG with an irregular pulse notification
*Guo et al.*	Huawei Watch GT	Detection of AF after irregular pulse notification	187,912	PPV (0.92) of AF with notification of ”suspected AF”
*Caillol et al.*	Apple Watch	Sensitivity and Specificity of Apple Watch for AF	256	Sensitivity: 96% Specificity: 91%
*Wasserlauf et al.*	Apple Watch with Kardia Band	Sensitivty to episode of AF	24	Sensitivity: 97%
*Drr et al.*	Samsung Gear Fit 2	Sensitivity, Specificity, and Accuracy of Samsung smartwatch to AF	508	Sensitivity: 94% Specificity: 98% Accuracy: 96%
*Al-Kaisey et al.*	Fitbit Charge HR, Apple Watch	Accuracy of HR in comparison to Holter	32	92–96% accurate HR, decrease in accuracy in AF

**Notes.**

PPV positive predicted value

Two studies have demonstrated that patients who use smartwatches have higher health utilization than patients who do not use wearables; however, many workups for these patients do not yield clinically significant results or diagnoses. In a study by Wyatt et al., only 11.4% of visit notes that included the words “Apple Watch” resulted in clinically actionable cardiovascular diagnoses^36^. 22% of the patients in the study had an existing diagnosis of AF, and 48.9% had a preexisting cardiovascular diagnosis.

Wyatt et al. found their number of patient evaluations needed to make one diagnosis of clinically actional cardiovascular disease was seven (95% confidence interval, 3.5–14.5) for symptomatic patients^36^. The study had a relatively small (*n* = 264) population and noted that the most common workup was a 12-lead ECG (59.8%)^36^. The cost of diagnostic testing was not included.

Wang et al. focused on patients with AF who used wearables, and paired those who did not use wearables by pulse rates^37^. They found that the mean composite use score, their measure of average healthcare utilization, was higher among individuals using wearables (3.55 [95% CI [3.31–3.80]] *vs* 3.27 [95% CI [3.14–3.40]]; *P* = .04)^37^, highlighting the potential harm of universal screening and the burden it could place on the healthcare system.

Despite their accuracy in the diagnosis of AF, smartwatches as screening tools are yet to demonstrate an increase in the detection of AF. A single study by Zimerman et al. concluded that while the FDA cleared the Apple Watch as a viable means to detect AF, there has been no change in the incidence of AF diagnoses in the 12 months following^38^. It is estimated that up to 18% of AF diagnoses are first detected only after stroke^39^. Raja et al. concluded that while wearable technology may not be the ultimate tool for the diagnosis of subclinical AF, it would not hurt as a guide or screener tool to monitor heart rhythm before talking to a doctor^39^. Under current FDA clearance, PPG can be used over the counter with the warning that “this device is not intended to provide a diagnosis”^40^. A modification to this clearance, approved in June of 2022, allowed these devices to expand and include a “atrial fibrillation history feature”, hinting at this device’s ability to translate health data to doctors’ offices at a later date^41^.

**Figure 2.**
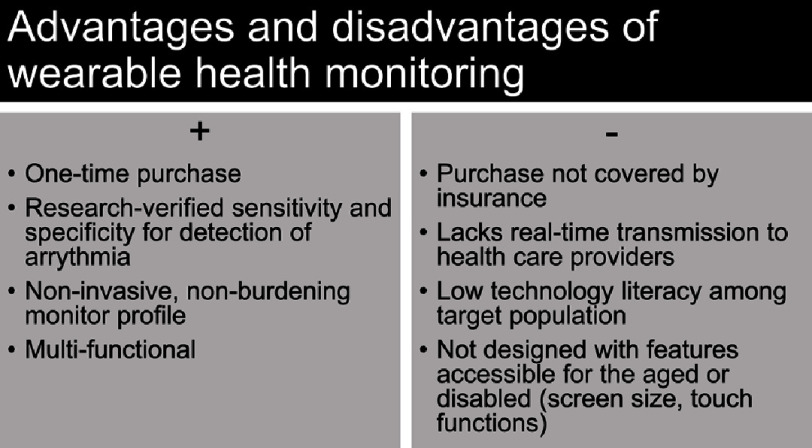
Textbox summary. A quick summary of the literature-identified advantages and disadvantages of using smartwatches for health monitoring.

Management strategies for AF revolve around anticoagulation needs and symptom control^42^. Once patients are anticoagulated, further medical control is determined based on subjective symptoms rather than on true rhythm monitoring. This leaves space for smartwatch utility as a monitor of arrhythmia burden, response to treatment, and confirmation of symptoms. A summary of the advantages and disadvantages of smartwatch use is presented in Figure 2.

## Conclusion

Atrial fibrillation and cardiac arrhythmias are serious medical concerns that can result in poor quality of life, syncope, and cerebrovascular accidents. The complications of undiagnosed arrhythmia result in a significant financial burden, and atrial fibrillation hospitalizations alone cost over US$4 billion. Recently, wearable smart devices have entered the market with the claim of providing medical-grade health monitoring for an affordable price. Studies of these devices’ capabilities have concluded that they are generally as accurate as traditional cardiac monitoring devices, and may act as both a sensitive and specific tool for future diagnosis. This could act as an affordable and available way for consumers to monitor their health and decrease the likelihood of CVA due to subclinical AF. Despite this, there are concerns regarding the accuracy of these devices in more active patients. Furthermore, these devices lack features catering to the elderly population, suffering from restraints of small screen size and interfaces built for younger generations. As these generations age, there may be increased functionality and practicality of smartwatches in the detection of atrial fibrillation. Finally, while these devices may assist in the detection of atrial fibrillation, further management is guided by functional and symptom status and thus does not require continuous cardiac monitoring.

## List of abbreviations

AF: Atrial Fibrillation
AC: Alternating Current
CVA: Cerebrovascular accident
DC: Direct current
PPG: Photoplethysmography
US: United States
